# FAMILY CAREGIVING COMPETENCIES FOR NURSING EDUCATION: COURSE AND CURRICULAR INTEGRATION

**DOI:** 10.1093/geroni/igae098.2412

**Published:** 2024-12-31

**Authors:** Sara Hart, Andra Davis, Kathryn Sexson, Hui Zhao, Tanya Seward, Connie Perkins, Nannette Cowen, Jennifer Mongoven

**Affiliations:** University of Utah, Salt Lake City, Utah, United States; University of Portland, Portland, Oregon, United States; University of California Davis, Davis, California, United States; James Madison University, Harrisonburg, Virginia, United States; University of Providence, South Great Falls, Montana, United States; St. Bonaventure University, St. Bonaventure, New York, United States; Binghamton University, Binghamton, New York, United States; University of California Davis, Davis, California, United States

## Abstract

The U.S. health care system is increasingly dependent on family caregivers who play an essential role in addressing the needs of loved ones who are aging, managing chronic or disabling conditions, or facing life-limiting illnesses. The National Consortium for Family Caregiving in Nursing Education has developed twenty entry-level nursing education competencies for identifying, integrating, and supporting family caregivers in person-centered and family-centered healthcare teams. These competencies fall within four domains: the Nature of Family Caregiving, Family Caregiving Identification and Assessment, Providing Family-centered Care, and the Context of Family Caregiving. Given the time and content limitations of nursing education and the dynamic nature of healthcare, new educational competencies often encounter resistance. Here we present the competencies, along side course and curricular resources and exemplars created by the Consortium. These resources are designed to support nursing educators in identifying and integrating content and teaching methods that advance the inclusion of family caregivers in patient and family-centered care teams and that promote nursing student development of family caregiving competencies.

